# Correction: MiR-198 represses tumor growth and metastasis in colorectal cancer by targeting fucosyl transferase 8

**DOI:** 10.1038/s41598-025-20205-4

**Published:** 2025-09-19

**Authors:** Minyu Wang, Jilin Wang, Xuan Kong, Huimin Chen, Yingchao Wang, Miao Qin, Yanwei Lin, Haoyan Chen, Jie Xu, Jie Hong, Ying-Xuan Chen, Weiping Zou, Jing-Yuan Fang

**Affiliations:** 1 State Key Laboratory for Oncogene and Related Genes, Key Laboratory of Gastroenterology & Hepatology, Ministry of Health, Division of Gastroenterology and Hepatology, Ren Ji Hospital, School of Medicine, Shanghai Jiao Tong University, Shanghai Cancer Institute, Shanghai Institute of Digestive Diseases, 145 Middle Shandong Road, Shanghai, 200001 China; 2 Department of Surgery, University of Michigan, Ann Arbor, MI 48109 USA

Correction to: *Scientific Reports* 10.1038/srep06145, published online 01 September 2014

This Article contains an error in Figure 4, panel J.

As a result of an error during figure preparation, the image used for the condition “HCT116/miR-198/FUT8 (0 h)” was a duplication of the condition “HCT116/NC (0 h)”. The authors have revisited the original data and provided a corrected version of the panel.

The correct Figure 4J and accompanying legend appear below.

**Fig. 4:**
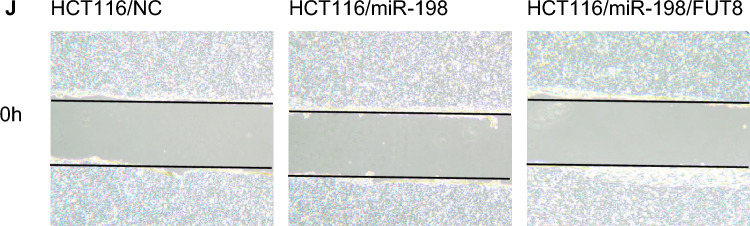
FUT8 may be involved in miR-198-mediated cell behavior. Fucosyl transferase 8 (FUT8) overexpression rescues miR-198-mediated inhibition of colorectal cancer cell proliferation, migration and invasion in vitro. SW1116 and HCT116 cells were stably transfected with a miR-198 overexpression plasmid or control negative plasmid and analyzed in CCK8 assays (**A**,**B**), transwell migration and invasion assays (**C**–**F**) and wound healing assays (**G**–**K**). Images are of representative fields for each experiment. Data in all statistical plots represent means ± SD.

